# Thiosinamine and Fibrolysin in Skin Affections

**Published:** 1909-02-27

**Authors:** 


### Dermatology.

### THIOSINAMINE AND FIBROLYSIN IN SKIN AFFECTIONS.

Thiosinamine, or allyl-sulpho-urea, is a substance prepared from oil of mustard by treating it with an alcoholic solution of ammonia. It was first introduced into therapeutics by Hebra in 1892 for lupus vulgaris. Although the drug seemed at first to promise favourable results in lupus, it did not stand the test of further experience. Some years later it was employed by other observers in the treatment of keloid and hypertrophic scars, and in these affections it has proved to be a valuable remedy. It has also been used in cases of sclerodermia. There are, however, many drawbacks to its employment. It was formerly given in a 15-per-cent. alcoholic solution, in doses of 0.2 c.c. up to 1 c.c., injected either into the lesion or at some other spot. These alcoholic injections were extremely painful, and an 8-per-cent. solution in glycerine and water (20 per cent. glycerine) was substituted, 15 minims being injected once or twice a week. But even this solution was found to give much pain and to cause a marked inflammatory reaction, and occasionally thiosinamine injections have caused necrosis. A great improvement has been more recently effected, at the suggestion of Mendel, in 1905, by which this drug has been rendered altogether non-irritant, and its administration therefore painless, and not followed by any severe inflammatory reaction. This modification consists in the combination of thiosinamine with salicylate of soda, and the product of this combination has been named "fibrolysin." Since the introduction of this modification the method of treatment has been much more extensively tested, and good results are reported in a very large number of affections in which excessive formation of fibrous tissue forms a part, such as Dupuytren's contraction, pyloric stenosis, post-operative abdominal adhesions, stricture of the urethra, opacity of the cornea, and many others. In dermatology fibrolysin has been largely employed in keloid, in hypertrophic scars after burns, in sclerodermia, and in elephantiasis. The most satisfactory method of administration is said to be by intramuscular injection into the gluteal region, but it may also be injected locally into the lesion. The dose of fibrolysin is 2.3 c.m. of a 15-per-cent. solution, given on alternate days, or at intervals of two to three days. An important technical point is to rinse the syringe in alcohol immediately after use, to prevent the accumulation of any exudate. In the case of hypertrophic scars and fibrous contractions after burns the fibrous tissue is dissolved and softened, and the tension relaxed. As regards sclerodermia the results have not been uniform, and further trials are necessary before it can be claimed as a reliable remedy in this condition. A case of von Recklinghausen's disease has been reported as much benefited, the fibrous nodules becoming softer and smaller. In elephantiasis from the deposit of fibrous tissue as a result of repeated attacks of cellulitis improvement has taken place in the direction of decrease in size of the affected parts.

The specific action of thiosinamine and of its modification, fibrolysin, in dissolving fibrous tissue is explained by the fact that these drugs lead to a temporary diminution of leucocytes, followed by a great excess of them, and consequent greater activity of lymph flow in the scar tissue, which softens and dissolves the connective tissue fibres.

